# Income Segregation, Conditional Cash Transfers, and Breast Cancer Mortality Among Women in Brazil

**DOI:** 10.1001/jamanetworkopen.2023.53100

**Published:** 2024-01-25

**Authors:** Joanna M. N. Guimarães, Julia M. Pescarini, J. Firmino de Sousa Filho, Andrea Ferreira, M. da Conceição C. de Almeida, Ligia Gabrielli, Isabel dos-Santos-Silva, Gervasio Santos, Mauricio L. Barreto, Estela M. L. Aquino

**Affiliations:** 1 Center for Data and Knowledge Integration for Health, Fiocruz, Salvador, Brazil; 2 Faculty of Epidemiology and Population Health, London School of Hygiene and Tropical Medicine, London, United Kingdom; 3 Ubuntu Center on Racism, Global Movements and Population Health Equity, Drexel University Dornsife School of Public Health, Philadelphia, Pennsylvania; 4 Gonçalo Moniz Institute, Fiocruz, Salvador, Brazil; 5 Secretaria de Saúde do Estado da Bahia, Centro de Diabetes e Endocrinologia da Bahia, Salvador, Brazil; 6 Instituto de Saúde Coletiva, Universidade Federal da Bahia, Salvador, Brazil

## Abstract

**Importance:**

Women living in income-segregated areas are less likely to receive adequate breast cancer care and access community resources, which may heighten breast cancer mortality risk.

**Objective:**

To investigate the association between income segregation and breast cancer mortality and whether this association is attenuated by receipt of the Bolsa Família program (BFP), the world’s largest conditional cash-transfer program.

**Design, Setting, and Participants:**

This cohort study was conducted using data from the 100 Million Brazilian Cohort, which were linked with nationwide mortality registries (2004-2015). Data were analyzed from December 2021 to June 2023**.** Study participants were women aged 18 to 100 years.

**Exposure:**

Women’s income segregation (high, medium, or low) at the municipality level was obtained using income data from the 2010 Brazilian census and assessed using dissimilarity index values in tertiles (low [0.01-0.25], medium [0.26-0.32], and high [0.33-0.73]).

**Main Outcomes and Measures:**

The main outcome was breast cancer mortality. Mortality rate ratios (MRRs) for the association of segregation with breast cancer deaths were estimated using Poisson regression adjusted for age, race, education, municipality area size, population density, area of residence (rural or urban), and year of enrollment. Multiplicative interactions of segregation and BFP receipt (yes or no) in the association with mortality (2004-2015) were assessed.

**Results:**

Data on 21 680 930 women (mean [SD] age, 36.1 [15.3] years) were analyzed. Breast cancer mortality was greater among women living in municipalities with high (adjusted MRR [aMRR], 1.18; 95% CI, 1.13-1.24) and medium (aMRR, 1.08; 95% CI, 1.03-1.12) compared with low segregation. Women who did not receive BFP had higher breast cancer mortality than BFP recipients (aMRR, 1.17; 95% CI, 1.12-1.22). By BFP strata, women who did not receive BFP and lived in municipalities with high income segregation had a 24% greater risk of death from breast cancer compared with those living in municipalities with low income segregation (aMRR, 1.24: 95% CI, 1.14-1.34); women who received BFP and were living in areas with high income segregation had a 13% higher risk of death from breast cancer compared with those living in municipalities with low income segregation (aMRR, 1.13; 95% CI, 1.07-1.19; *P* for interaction = .008). Stratified by the amount of time receiving the benefit, segregation (high vs low) was associated with an increase in mortality risk for women receiving BFP for less time but not for those receiving it for more time (<4 years: aMRR, 1.16; 95% CI, 1.07-1.27; 4-11 years: aMRR, 1.09; 95% CI, 1.00-1.17; *P* for interaction <.001).

**Conclusions and Relevance:**

These findings suggest that place-based inequities in breast cancer mortality associated with income segregation may be mitigated with BFP receipt, possibly via improved income and access to preventive cancer care services among women, which may be associated with early detection and treatment and ultimately reduced mortality.

## Key Points

**Question:**

Is income segregation associated with breast cancer mortality, and is this association attenuated by the Bolsa Família program (BFP), a conditional cash-transfer program?

**Findings:**

In this cohort study of 21 680 930 Brazilian women across municipalities with low, medium, and high income segregation, increased segregation was associated with increased risk of breast cancer mortality in a dose-response pattern. Moreover, the magnitude of the association of segregation with mortality was significantly lower for women who received BFP compared with women who did not receive BFP.

**Meaning:**

These findings suggest the importance of social policies, such as the BFP, in reducing health inequities, with policy implications (eg, including breast cancer screening in BFP conditionalities to increase breast cancer early detection).

## Teaser

This cohort study investigates the association of income segregation with breast cancer mortality and the interaction of a cash-transfer program with this association among women in Brazil.

## Introduction

Breast cancer is the leading cause of cancer deaths among women worldwide.^1^ The burden of breast cancer mortality is not evenly distributed across places or residential areas.^2,3,4^ Place-based inequities in breast cancer outcomes are due to structural and environmental factors that shape people’s behaviors and life conditions and limit access to effective screening, diagnosis, and treatment services.^2,3,5^ Unhealthy food environments, greater exposure to harmful pollutants, and tobacco marketing may affect cancer risk through constraints on individual behaviors (eg, diet, smoking, and physical activity) or through mechanisms involving stress.^6^ Evidence shows that living in economically disadvantaged areas is associated with poorer access to screening and care and unfavorable breast cancer outcomes, such as late-stage diagnosis, less adequate treatment, poorer survival, and mortality.^2,3,4,5,7^

Income residential segregation (hereafter *income segregation*) is defined as the systematic separation of individuals into different geographic areas based on their income^8^ due to discriminatory housing practices and policies that historically marginalize individuals with the lowest incomes.^4,8,9^ Income segregation operates as a fundamental cause of health inequities because it reinforces sociospatial differences in accessing health-promoting resources and ultimately leads to differences in individual health.^9^ Women who live in more segregated areas are less likely to receive adequate breast cancer care through its continuum (ie, from primary prevention to early detection, such as mammographic screening, and diagnosis to early treatment and rehabilitation).^2,10,11,12^ They are also less likely to access other community resources (eg, education and transportation) or engage in health-related behaviors, which are known to influence breast cancer incidence, survival, and mortality.^2,4,5,7,10^ According to Krieger et al (2020),^4^ neighborhood factors, including the inability to access health care and inadequate transportation, are associated with the stage of diagnosis and biological embodiment of cancer risk.

Implemented in 2004, Brazil’s Bolsa Família program (BFP) is the world’s largest conditional cash-transfer program, targeting low and extremely low income households.^13^ Aimed at reducing poverty and inequality, improving nutritional status, and increasing access to preventive health services and education, the BFP requires beneficiaries to comply with conditions, such as prenatal visits for pregnant women, vaccination for children, and minimum school attendance by children.^13,14,15^ Nearly 90% of BFP beneficiary families have women as recipients.^16^ Evidence shows that conditional cash-transfer programs are effective, associated with increases in women’s use of preventive health care services (eg, Papanicolaou test tests),^14^ improved health status,^17^ and enhanced empowerment among women upon increasing familial income.^15^ Individual-level income may attenuate the association between area-level socioeconomic disadvantage and breast cancer mortality.^7^ Therefore, we hypothesized that the BFP would be associated with improved access among women to community resources and preventive cancer care services, such as clinical breast examinations and mammographic screening, and thus early detection and treatment and ultimately reductions in the breast cancer mortality associated with income segregation.

This study addresses some critical gaps. First, it examines the association of a structural contextual factor, income segregation, with breast cancer mortality rather than focusing on individual attributes, which may provide a more comprehensive range of intervention strategies beyond the individual. Second, studies on the association of the BFP with health inequities have been focused on child health outcomes,^15,18,19^ and women’s health has been less explored. Using individual data from a large-scale, population-based cohort in Brazil, which includes more than half of the Brazilian population, we investigated the association between income segregation and breast cancer mortality, the association between being a recipient of BFP and breast cancer mortality, and the interaction between income segregation and being a recipient of BFP in the association with breast cancer mortality.

## Methods

This cohort study was approved by the Ethics Committee from the Instituto Gonçalo Moniz–Oswaldo Cruz Foundation with a waiver of informed consent because data were obtained from secondary datasets. This study followed the Strengthening the Reporting of Observational Studies in Epidemiology (STROBE) reporting guideline.

### Study Design and Participants

This is a longitudinal study conducted within the 100 Million Brazilian Cohort, a population-based cohort assembled from the Brazilian Government Unified Register for Social Programmes (CadÚnico), which includes data from more than 114 million Brazilians with low incomes (nearly 55% of the country’s population) for the 2001 to 2015 period.^19^ To be registered in CadÚnico, families are required to apply and have an income of up to half of the minimum wage per capita (approximately $125 US in 2020) or a total family income of up to 3 times the minimum wage (approximately $750 US).^19^ For this study, the 100 Million Brazilian Cohort baseline data set was probabilistically linked to the Brazilian Mortality Database^20^ to identify women in the cohort who had died from breast cancer during follow-up. Detailed linkage procedures can be found elsewhere.^19,21^

**Figure 1.**
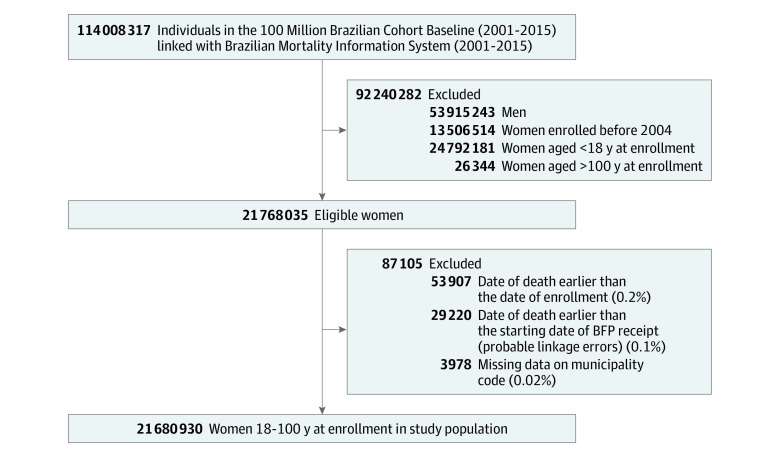
Study Flowchart BFP indicates Bolsa Família program.

All women enrolled in CadÚnico between January 1, 2004 (given that levels of data missingness were high for those enrolled in previous years), and December 31, 2015 (the last year for which mortality data were available), and who were aged between 18 and 100 years at enrollment were potentially eligible to participate in the study. Women whose date of death was earlier than their date of enrollment into the cohort or earlier than the starting date of BFP receipt and those with missing data on municipality code (likely due to linkage errors) were excluded, leaving 21 680 930 women for analysis (Figure 1). Women’s follow-up was determined by the baseline enrollment in CadÚnico (2004-2015) up to the occurrence of death or the end of the study. Data were analyzed from December 2021 to June 2023.

### Measures

#### Exposure

Income segregation was obtained using income data by census tract from the 2010 Brazilian census^22^ and was assessed at the municipality level using the dissimilarity index, which measures evenness and indicates the percentage of a population group that would have to change residence to achieve total integration.^23^ The index ranges from 0 (complete integration) to 1 (complete segregation) and was calculated for each Brazilian municipality according to the following formula:
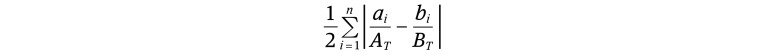
where *n* is the number of census tracts in a municipality; *A_T_* and *B_T_* are the percentage of households with a mean per capita income of less than or equal to one-half and more than one-half of the minimum wage in that municipality, respectively; and *a_i_* and *b_i_* are their respective populations in census tract *i*. Segregation was categorized into tertiles for analysis (low [0.01-0.25], medium [0.26-0.32], and high [0.33-0.73]).

#### Outcome

Individual information on the occurrence of deaths during follow-up, including dates and their underlying cause coded according to the *International Statistical Classification of Diseases and Related Health Problems, Tenth Revision* (*ICD-10*), was obtained through linkage to the National Mortality Database.^20^ Deaths from breast cancer were those with an underlying cause coded as C50 according to *ICD-10*.

#### Covariates

Data on covariates were also collected at enrollment into CadÚnico, including individual information on age, education level (categorized as ≤5, 6-9, and >9 years of schooling), and year of participants' enrollment in CadÚnico (2004-2015). Self-reported race (Asian, Black, Indigenous, Parda, or White) was assessed because it may potentially confound the associations of segregation and BFP with mortality. The term *Parda* is used to denote multiracial individuals and may also include those of mostly Indigenous ancestry.^24^ Municipality-level data included the municipality area size (in kilometers squared), population density (municipality population at 2010 census/municipality area size), and area of residence (rural vs urban).

#### Effect Modifier

The BFP variable was defined on whether the participant was a recipient (yes or no) after enrollment in CadÚnico. BFP recipients were also classified according to the time in years receiving the benefit, calculated based on the starting and ending date of each participant BFP receipt (4-11 years, <4 years, no receipt). We considered that when individuals started receiving BFP they continued receiving it for the remaining study period because only 0.3% of BFP recipients in our cohort stopped before the end of follow-up and ancillary benefits (ie, increased access to the Brazilian Universal Healthcare system and education) are expected to continue after the end of the cash benefit.^13^

### Statistical Analysis

Sample characteristics were described across income segregation tertiles, and differences were tested using χ^2^ tests and analysis of variance; sample characteristics were also described across BFP receipt groups (yes or no) (eTable 1 in Supplement 1). Person-time at risk was calculated from the time a woman was enrolled into CadÚnico to the time of her death from breast cancer, the time of her death from another cause, or the end of follow-up (for this analysis, December 31, 2015), whichever occurred first. Individual-level Poisson regression models were used to estimate mortality rate ratios (MRRs) and 95% CIs. Associations of income segregation with breast cancer mortality were estimated without adjustment (model 1) and after adjustments for individual age, race, and education (model 2) because these factors may confound income segregation differences in breast cancer mortality. Then, we added municipality-level variables area size, population density, and area of residence and the individual’s year of enrollment to model 2 (model 3) to account for differences in these variables by segregation levels. Model 4 added the BFP receipt variable to estimate the association of being a recipient of the BFP with breast cancer mortality (conceptual model; eFigure 1 in Supplement 1).

To investigate whether BFP receipt modified the association between income segregation and breast cancer deaths, a multiplicative interaction term between income segregation and BFP receipt was added to Model 4. If the interaction term was significant (2-sided *P* < .05) based on likelihood ratio test, stratified effects were obtained by calculating the MRR for income segregation within strata of BFP receipt. We additionally explored whether the time in years receiving the BFP modified the association between segregation and breast cancer mortality, following the same steps of interaction analysis described previously.

In sensitivity analysis, to assess the extent to which the observed association of income segregation with mortality may reflect the poorer quality of the data (ie, underreporting of mortality among individuals with the lowest incomes), we restricted the analysis to Brazilian municipalities known to have high death registration coverage (≥95%). All statistical analyses were performed in Stata statistical software version 15.1 (StataCorp).

## Results

Among 21 680 930 studied women (mean [SD] age, 36.1 [15.3] years; 96 085 Asian [0.4%], 1 772 843 Black [8.2%], 11 549 000 Parda [53.3%], 104 252 Indigenous [0.5%], and 7 110 375 White [32.8%]), there were 7 227 998 women, 7 309 565 women, and 7 143 367 women living in municipalities with low, medium, and high income segregation, respectively. The incidence of breast cancer mortality was 4625 women (0.064%), 4904 women (0.067%), and 5858 women (0.082%) among those living in Brazilian municipalities with low, medium, and high income segregation, respectively (Table 1). Women living in municipalities with high income segregation were more likely to be older, Indigenous or Black, and have a higher education level and less likely to be BFP recipients compared with those living in municipalities with medium or low segregation. Municipalities with more income segregation were larger, had higher population density, and had larger proportions of women residing in urban areas than municipalities with less income segregation. When comparing women who received the BFP with women who did not receive the BFP, nonrecipients were more likely to be White, older, and higher educated and tended to live in more urban and segregated municipalities than BFP recipients (eTable 1 in Supplement 1).

**Table 1.** Study Population Characteristics by Income Segregation Tertiles^a^

Characteristic	Participants, No. (%)				
	Overall (N = 21 680 930)	Low income segregation (n = 7 227 998)^b^	Medium income segregation (n = 7 309 565)^b^	High income segregation (n = 7 143 367)^b^	*P* value
Breast cancer deaths	15 387 (0.071)	4625 (0.064)	4904 (0.067)	5858 (0.082)	<.001
Missing values	0	0	0	0	
Age at baseline, mean (SD), y	36.1 (15.3)	35.5 (15.6)	36.1 (15.5)	36.6 (14.9)	<.001
Missing values	0	0	0	0	
Self-declared race					
Asian	96 085 (0.4)	32 381 (33.7)	32 957 (34.3)	30 747 (32.0)	<.001
Black	1 772 843 (8.2)	549 581 (31.0)	537 171 (30.3)	686 091 (38.7)	
Indigenous	104.252 (0.5)	19 391 (18.6)	27 105 (26.0)	57 756 (55.4)	
Parda	11 549 000 (53.3)	3 995 954 (34.6)	3 892 013 (33.7)	3 661 033 (31.7)	
White	7 110 375 (32.8)	2 317 98 (32.6)	2 467 300 (34.7)	2 325 093 (32.7)	
Missing values	1 048 375 (4.8)	314 512 (30.0)	352 254 (33.6)	381 609 (36.4)	
Education, y					
>9	5 361 078 (24.7)	1 543 989 (28.8)	1 801 322 (33.6)	2 015 765 (37.6)	<.001
6-9	5 266 803 (24.3)	1 732 779 (32.9)	1 753 845 (33.3)	2 015 765 (33.8)	
≤5	8 215 291 (37.9)	2 998 581 (36.5)	2 801 414 (34.1)	2 415 296 (29.4)	
Missing values	2 837 758 (13.1)	956 324 (33.7)	953 487 (33.6)	927 947 (32.7)	
BFP recipient^c^					
No	5 962 519 (27.5)	1 836 456 (30.8)	2 128 619 (35.7)	1 997 444 (33.5)	<.001
Yes	15 718 411 (72.5)	5 391 415 (34.3)	5 187 076 (33.0)	5 139 920 (32.7)	
Missing values	0	0	0	0	
Municipality area, mean (SD), km^2^	2269.9 (7193.5)	1141.1 (3067.8)	2803.9 (8455.8)	2865.7 (8510.7)	<.001
Missing values	0	0	0	0	
Population density, mean (SD) No. of inhabitants/km^2^^d^	1342.6 (2464.9)	639.7 (1910.9)	473.9 (1142.6)	2942.6 (3094.8)	<.001
Missing values	0	0	0	0	
Area of residence					
Urban	17 327 758 (80.0)	5 025 050 (29.0)	5 856 782 (33.8)	6 445 926 (37.2)	<.001
Rural	4 236 786 (19.5)	2 169 235 (51.2)	1 402 376 (33.1)	665 175 (15.7)	
Missing values	116 386 (0.5)	40 037 (34.4)	40 735 (35.0)	35 614 (30.6)	

Abbreviation: BFP, Bolsa Família program.

^a^ Data are from the 100 Million Brazilian Cohort (2004-2015), including 21 680 930 women aged 18 to 100 years.

^b^ Income segregation was measured using the per capita household income–based dissimilarity index: one-half the minimum wage or less vs more than one-half the minimum wage in tertiles (low [0.01-0.25], medium [0.26-0.32], and high [0.33-0.73]).

^c^ Categorized by whether the participant was a BFP recipient.

^d^ Measured at the municipality level.

Age-standardized breast cancer mortality rates per 100 000 women-years in the overall cohort were highest (9.4 deaths; 95% CI, 9.2-9.7 deaths), intermediate (7.4 deaths; 95% CI, 7.2-7.6 deaths), and lowest (6.7 deaths; 95% CI, 6.5-6.9 deaths) for women living in municipalities with high, medium, and low income segregation, respectively (eFigure 2 in Supplement 1). By BFP recipient groups (yes or no), the positive association of increased segregation levels with mortality rates persisted for both BFP groups, although women not receiving BFP had higher rates than those receiving BFP (eFigure 2 in Supplement 1).

After adjustments for age, race, and education (model 2), MRRs were 1.38 (95% CI, 1.32-1.44) and 1.08 (95% CI, 1.03-1.13) for women living in municipalities with high and medium segregation, respectively, compared with women from municipalities with low segregation (Table 2). After additional adjustments for municipality area size, population density, area of residence, and year of enrollment (model 3), the MRR was attenuated for women from highly segregated municipalities by approximately one-half (MRR, 1.18; 95% CI, 1.12-1.23), but important differences remained. Further adjustment for BFP receipt (model 4) did not change associations for women from municipalities with high (MRR, 1.18; 95% CI, 1.13-1.24) or medium (MRR, 1.08; 95% CI, 1.03-1.12) vs low segregation. After full adjustments (model 4), women not receiving BFP had a 17% higher risk of breast cancer mortality than BFP recipients (MRR, 1.17; 95% CI, 1.12-1.22). Black women had a 10% higher risk of dying from breast cancer than White women (MRR, 1.10; 95% CI, 1.04-1.17) (model 4).

**Table 2.** Association of Income Segregation in Tertiles With Breast Cancer Mortality

Factor	MRR (95% CI)^a^^,^^b^		
	Model 2	Model 3	Model 4
Income segregation^c^			
Medium vs low	1.08 (1.03-1.13)	1.08 (1.03-1.12)	1.08 (1.03-1.12)
High vs low	1.38 (1.32-1.44)	1.18 (1.12-1.23)	1.18 (1.13-1.24)
Age at baseline per 1-y increase	1.05 (1.05-1.06)	1.05 (1.05-1.06)	1.05 (1.05-1.05)
Race			
Asian vs White	0.71 (0.53-0.96)	0.73 (0.54-0.98)	0.73 (0.54-0.98)
Black vs White	1.11 (1.05-1.17)	1.09 (1.03-1.15)	1.10 (1.04-1.17)
Parda vs White	0.84 (0.81-0.88)	0.86 (0.83-0.89)	0.87 (0.84-0.90)
Indigenous vs White	0.45 (0.32-0.63)	0.62 (0.44-0.87)	0.64 (0.45-0.90)
Education			
6-9 y vs >9 y	1.06 (1.00-1.12)	1.05 (0.99-1.12)	1.06 (1.00-1.13)
≤5 y vs >9 y	0.92 (0.87-0.97)	0.97 (0.91-1.03)	0.98 (0.93-1.04)
Municipality size per 1-unit (km^2^) increase	NA	1.00 (1.00-1.00)	1.00 (1.00-1.00)
Municipality population density per 1-unit (No. of inhabitants/km^2^) increase	NA	1.00 (1.00-1.00)	1.00 (1.00-1.00)
Area of residence: rural vs urban	NA	0.70 (0.66-0.74)	0.70 (0.67-0.74)
Year of enrollment per 1-y increase	NA	0.99 (0.99-1.00)	0.98 (0.98-0.99)
BFP recipient: no vs yes	NA	NA	1.17 (1.12-1.22)

Abbreviations: BFP, Bolsa Família program; MRR, mortality rate ratio; NA, not applicable.

^a^ Data are from the 100 Million Brazilian Cohort (2004-2015), including 21 680 930 women aged 18 to 100 years.

^b^ Model 1 was a crude analysis (medium vs low segregation: MRR, 1.12; 95% CI, 1.07-1.16; high vs low segregation: MRR, 1.45; 95% CI, 1.39-1.51). Model 2 adjusted for age, race, and education. Model 3 included model 2 adjustments and adjustments for municipality area size, municipality population density, area of residence, and year of enrollment. Model 4 included model 3 adjustments and adjusted for BFP receipt.

^c^ Income segregation was measured using the dissimilarity index in tertiles: low (0.01-0.25), medium (0.26-0.32), and high (0.33-0.73).

**Figure 2.**
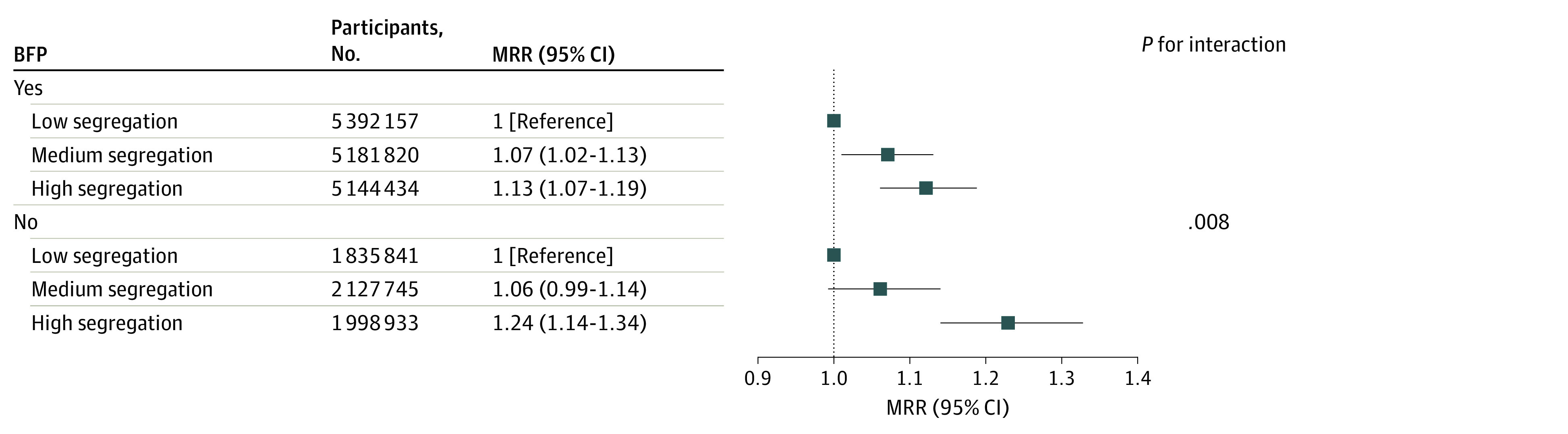
Association of Income Segregation With Breast Cancer Mortality The association is shown by income segregation, measured using the dissimilarity index in tertiles (low, medium, and high), stratified by Bolsa Família program (BFP) receipt. Data are from the 100 Million Brazilian Cohort (2004-2015), including 21 680 930 women aged 18 to 100 years. Outcome was adjusted for age, race, education, municipality area size, municipality population density, area of residence (rural or urban), year of enrollment, and interaction term (income segregation × BFP receipt). MRR indicates mortality rate ratio.

There was evidence of a multiplicative interaction between income segregation and BFP receipt, suggesting that the association between segregation and breast cancer deaths was different for women who received and did not receive BFP (*P* interaction = .008) (Figure 2). By BFP receipt strata, among women who received BFP, those living in municipalities with high segregation had a 13% greater risk of dying from breast cancer compared with those living in municipalities with low segregation (MRR, 1.13: 95% CI, 1.07-1.19); among women who did not receive BFP, the risk of dying for those living in municipalities with high segregation was 24% higher compared with those living in municipalities with low segregation, and 95% CIs virtually did not overlap (MRR, 1.24: 95% CI, 1.14-1.34) (Figure 2). Stratification by the time in years receiving the BFP benefit showed that income segregation (high vs low) was associated with mortality only among women receiving the benefit for less time (<4 years: MRR, 1.16; 95% CI, 1.07-1.27; 4-11 years: MRR, 1.09; 95% CI, 1.00-1.17) (*P* for interaction <.001) (Table 3).

**Table 3.** Association of Income Segregation in Tertiles With Breast Cancer Mortality by BFP Duration^a^^,^^b^

Income segregation^c^	MRR (95%CI)
**BFP received ≥4 y (n = 12 606 997)**	
Medium vs low	1.08 (1.00-1.16)
High vs low	1.09 (1.00-1.17)
**BFP received, <4 y (n = 3 111 414)**	
Medium vs low	1.15 (1.06-1.25)
High vs low	1.16 (1.07-1.27)
**BFP not received (n = 5 962 519)**	
Medium vs low	1.06 (0.99-1.14)
High vs low	1.24 (1.14-1.34)

Abbreviations: BFP, Bolsa Família program; MRR, mortality rate ratio.

^a^ Data are from the 100 Million Brazilian Cohort (2004-2015), including 21 680 930 women aged 18-100 years.

^b^ Adjusted for age, race, education, municipality area size, municipality population density, area of residence (rural or urban), year of enrollment, and interaction term (income segregation × BFP duration); *P* for multiplicative interaction <.001.

^c^ Income segregation was measured using the dissimilarity index in tertiles: low (0.01-0.25), medium (0.26-0.32), and high (0.33-0.73). All outcomes use low income segregation as the reference group.

A sensitivity analysis restricted to Brazilian municipalities with high (≥95%) death registration coverage yielded similar overall findings. However, the MRR was decreased for high vs low income segregation (MRR for all municipalities, 1.18; 95% CI, 1.13-1.24; MRR for municipalities with high death registration coverage, 1.14; 95% CI, 1.07-1.21) and for no BFP receipt vs BFP receipt (MRR for all municipalities, 1.17: 95% CI, 1.12-1.22; MRR for municipalities with high death registration coverage, 1.11; 95% CI, 1.05-1.18) (eTable 3 in Supplement 1).

## Discussion

To our knowledge, this cohort study is the first study to investigate a possible interaction of a conditional cash-transfer program (the BFP) in the association of income segregation with breast cancer mortality. Moreover, to our knowledge, no study has evaluated the association of the BFP with breast cancer mortality. This cohort study using individual data of more than 20 million Brazilian women across municipalities with low, medium and high income segregation found that greater segregation was associated with increased risk among women of dying from breast cancer in a dose-response pattern. We also found that women not receiving the BFP had a higher risk of breast cancer death than BFP recipients and the association between segregation and mortality varied significantly by BFP receipt strata. Women living in highly segregated areas who did not receive the BFP had a 24% higher risk of dying from breast cancer compared with a 13% higher risk for women living in highly segregated areas who received the BFP. Moreover, there was an association between segregation and mortality only for women receiving the benefit for less time.

Our study demonstrated that income segregation was associated with increased individual breast cancer mortality. This is consistent with results from empirical^11,12,25,26,27^ and review studies,^3,5,28^ although that evidence comes from the US and most studies have focused on racial^11,12,25,26,27^ rather than income^25,26^ segregation. According to these studies, higher breast cancer mortality in more vs less segregated areas was mainly associated with inadequate access to quality cancer care and differential screening access (eg, poorer mammography use), which was associated with a later stage at diagnosis and poorer treatment. For example, Haas et al (2008)^12^ found that women with breast cancer who resided in more vs less segregated municipalities had 27% lower odds of receiving adequate breast cancer care. Dai (2010)^11^ showed that highly segregated areas and poor mammography access were associated with high late-stage breast cancer diagnosis rates. In addition, women living in areas of high vs low redlining (ie, the systematic denial of mortgages in the US based on place of residence) had increased rates of breast cancer mortality^2^ and were more likely to have a stage IV breast cancer diagnosis.^2,4^ In our study, women who resided in municipalities with more income segregation were more likely to live in municipalities that were urban, larger, and had higher population density, which correlates with large social and health inequalities,^6,28^ contributing to disparities in access to primary health care, as well as breast cancer early detection and mortality. Our study also found that Black women had a 10% higher risk of breast cancer mortality than White women, which is consistent with previous Brazilian research^29,30^ and may be explained by barriers to accessing health care and discrimination shaped by structural racism. This may be potentially magnified by living in more segregated areas^31^ and deserves further exploration.

Women who did not receive BFP showed a 17% higher risk of dying from breast cancer than BFP recipients. The positive association of the BFP with health has been demonstrated in other Brazilian studies, including reductions in perinatal outcomes,^15,32^ child^18,33^ and maternal mortality,^34,35^ suicide rates,^36^ cardiovascular diseases,^13^ and communicable diseases, such as leprosy^37^ and tuberculosis.^38,39^ However, no studies to our knowledge have investigated the association of the BFP or any other cash-transfer program with breast cancer outcomes or mortality. The BFP may be associated with reduced risk among women of dying from breast cancer in 2 ways. First, it may improve income, which could enhance women’s access to medications, quality nutrition, and other behaviors related to health promotion, and may also relieve psychological barriers to health. Second, although BFP conditionalities do not comprise the use of preventive cancer care services, they impose a minimum usage of health services for women’s health, promote health education, and improve women’s general health status.^13,14,34,35,40^

We found that the BFP interacted with income segregation such that BFP attenuated the association of segregation with breast cancer mortality. In other words, receiving the BFP was associated with mitigation of the detrimental factors of living in more segregated areas associated with breast cancer mortality risk. Moreover, for women receiving the BFP for less time (<4 years), there was an association between segregation and mortality, while there was no association for women receiving it for more time (4-11 years), which may strengthen our findings. Our results may be explained because the BFP is associated with reductions in socioeconomic inequalities reinforced by poor residential contexts and expanded access to health care among women.^13,14,34,35^ Conditional cash transfers can make women living in highly segregated areas less vulnerable to health-damaging physical and social environments due to increased access to goods and basic social services. The BFP facilitates health care access, which may be positively associated with breast cancer stage at diagnosis and treatment. Cash transfers may also be associated with improved access to transportation among women, allowing them to seek quality health care in other settings. In addition, conditional cash-transfer programs support women’s sense of empowerment and strengthen their self-esteem and access to rights.^14,34,41,42^

Our study’s strengths include the very large sample size of more than 20 million women, important when investigating rare events, such as deaths from breast cancer, especially when testing interactions. Another strength is the wealth of data on social determinants of breast cancer mortality. Moreover, the long follow-up period of more than 10 years enabled us to evaluate outcomes associated with the BFP in a long-term perspective, such as by stratifying the analysis by number of years receiving the benefit. In addition, sensitivity analysis restricted to municipalities with high death registration coverage yielded similar results. Thus, an information bias due to outcome misclassification is unlikely to explain our findings.

### Limitations

This study has several limitations. The 100 Million Brazilian Cohort comprises the lowest income half of the population who applied for social protection programs. Therefore, our study is generalizable to this population of Brazilian women. We did not perform a causal analysis, and this is an observational study, so no causal conclusions can be made. Unfortunately, we lacked data on access to health care (eg, access to preventive cancer care services, mammography use, breast cancer stage at diagnosis, and treatment). Future research is needed to empirically assess the role of health care access in the associations of income segregation and BFP with breast cancer mortality. Moreover, additional work examining income and racial segregation is also needed to better understand the associations of poverty and racism with health in the Brazilian context.

## Conclusions

This cohort study found that the BFP was associated with reductions in the place-based inequities in breast cancer mortality associated with income segregation, possibly by improving women’s income and access to preventive cancer care services, leading to early detection and treatment and ultimately reducing mortality. In the context of a large, middle-income country, our study adds novel insight into how social environments may be associated with breast cancer mortality and draws attention to the importance of social policies, such as conditional cash-transfer programs, in promoting health and reducing health inequities. We suggest inclusion of breast cancer early detection through regular clinical breast examination coupled with screening (eg, mammography) to BFP conditionalities, as has been done in other Latin American countries.^41^

## Notes

Supplement 1. **eTable 1.** Characteristics of Sample by Bolsa Família Program Receipt **eFigure 1.** Conceptual Model for the Interaction of Income Segregation and Bolsa Família Program Receipt in the Association With Breast Cancer Mortality **eFigure 2.** Breast Cancer Age-Standardized Mortality Rates Per 100 000 Women-Years by Income Segregation Tertiles and Bolsa Família Program Receipt Groups **eTable 2.** Mortality Rate Ratios and 95% CIs From Sensitivity Analysis to Examine Low Death Registration Rate Across Brazilian Municipalities and the Association Between Income Segregation in Tertiles and Breast Cancer Mortality

Supplement 2.
Data Sharing Statement
