# Supplementary material for: Income Segregation, Conditional Cash Transfers, and Breast Cancer Mortality Among Women in Brazil
Source: JAMA Netw Open. 2024 Jan 25;7(1):e2353100. doi: 10.1001/jamanetworkopen.2023.53100 (PMC10811554; doi:10.1001/jamanetworkopen.2023.53100)
Supplement: Supplement 1. — eTable 1. Characteristics of Sample by Bolsa Família Program Receipt eFigure 1. Conceptual Model for the Interaction of Income Segregation and Bolsa Família Program Receipt in the Association With Breast Cancer Mortality eFigure 2. Breast Cancer Age-Standardized Mortality Rates Per 100 000 Women-Years by Income Segregation Tertiles and Bolsa Família Program Receipt Groups eTable 2. Mortality Rate Ratios and 95% CIs From Sensitivity Analysis to Examine Low Death Registration Rate Across Brazilian Municipalities and the Association Between Income Segregation in Tertiles and Breast Cancer Mortality [file jamanetwopen-e2353100-s001.pdf]

Supplemental Online Content

Guimarães JMN, Pescarini JM, Sousa Filho JFD, et al. Income segregation, conditional cash transfers, and breast cancer mortality among women in Brazil. *JAMA Netw Open*. 2024;7(1):e2353100. doi:10.1001/jamanetworkopen.2023.53100

eTable 1. Characteristics of Sample by Bolsa Família Program Receipt

eFigure 1. Conceptual Model for the Interaction of Income Segregation and Bolsa Família Program Receipt in the Association With Breast Cancer Mortality

eFigure 2. Breast Cancer Age-Standardized Mortality Rates Per 100 000 Women-Years by Income Segregation Tertiles and Bolsa Família Program Receipt Groups

eTable 2. Mortality Rate Ratios and 95% CIs From Sensitivity Analysis to Examine Low Death Registration Rate Across Brazilian Municipalities and the Association Between Income Segregation in Tertiles and Breast Cancer Mortality

This supplemental material has been provided by the authors to give readers additional information about their work.

© 2023 Guimarães JMN et al. *JAMA Network Open.*

eTable 1. Characteristics of the sample, by Bolsa Família Programme (BFP) recipient. [a]

Variables	Overall<br>n=21,680,930	Bolsa Família recipient [b], %<br>Yes<br>n=15,718,411	No<br>n=5,962,519	*P*-value
Breast cancer deaths, n (%)	15,387 (0.071)	0.063	0.092	< 0.001
Missing values	0	0	0	
Age at baseline, mean (SD)	36.1 (15.3)	32.3 (12.1)	46.1 (18.5)	< 0.001
Missing values	0	0	0	
Self-declared race				
White	7,110,375	64.9	35.1	< 0.001
Parda	11,549,000	76.7	23.3	
Black	1,772,843	78.1	21.9	
Asian	96,085	63.6	26.4	
Indigenous	104,252	90.9	9.1	
Missing values	1,048,375	66.8	33.2	
Education in years				
>9	5,361,078	61.6	38.4	< 0.001
6-9	5,266,803	82.8	17.2	
<=5	8,215,291	68.6	31.4	
Missing values	2,837,758	85.2	14.8	
Income segregation [c]				
Low	7,227,998	74.6	25.4	< 0.001
Medium	7,309,565	70.9	29.1	
High	7,143,367	72.0	28.0	
Missing values	0	0	0	
Municipality area in km2 [d], mean (SD)	2269.9 (7193.5)	2382.8 (7703.6)	1972.3 (5620.7)	< 0.001
Missing values	0	0	0	
Population density [d], mean (SD)	1342.6 (2464.9)	1341.2 (2460.5)	1346.1 (2476.6)	< 0.001
Missing values	0	0	0	
Area of residence				
Urban	17,327,758	70.1	29.9	
Rural	4,236,786	82.5	17.5	< 0.001
Missing values	116,386	67.0	33.0	

[a] Data are from the 100 Million Brazilian Cohort (2004-2015), n=21,680,930 women aged 18-100 years.
[b] Whether the participant was a Bolsa Família Programme (BFP) recipient.
[c] Percapita household income-based dissimilarity index: ≤1/2 minimum wage vs >1/2 minimum wage, in tertiles.
[d] Measured at the municipality level.

© 2023 Guimarães JMN et al. *JAMA Network Open.*

eFigure 1. Conceptual Model for the Interaction of Income Segregation and Bolsa Família Program Receipt in the Association With Breast Cancer Mortality

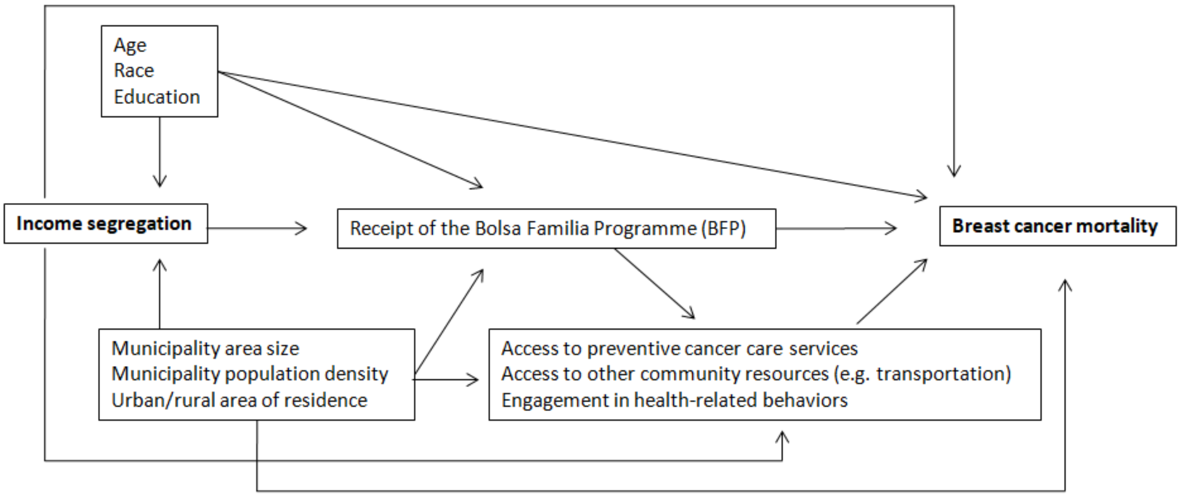

© 2023 Guimarães JMN et al. *JAMA Network Open.*

eFigure 2. Breast cancer age-standardized[a] mortality rates, per 100,000 women-years, by income segregation tertiles and Bolsa Família Programme (BFP) recipient groups.[b]

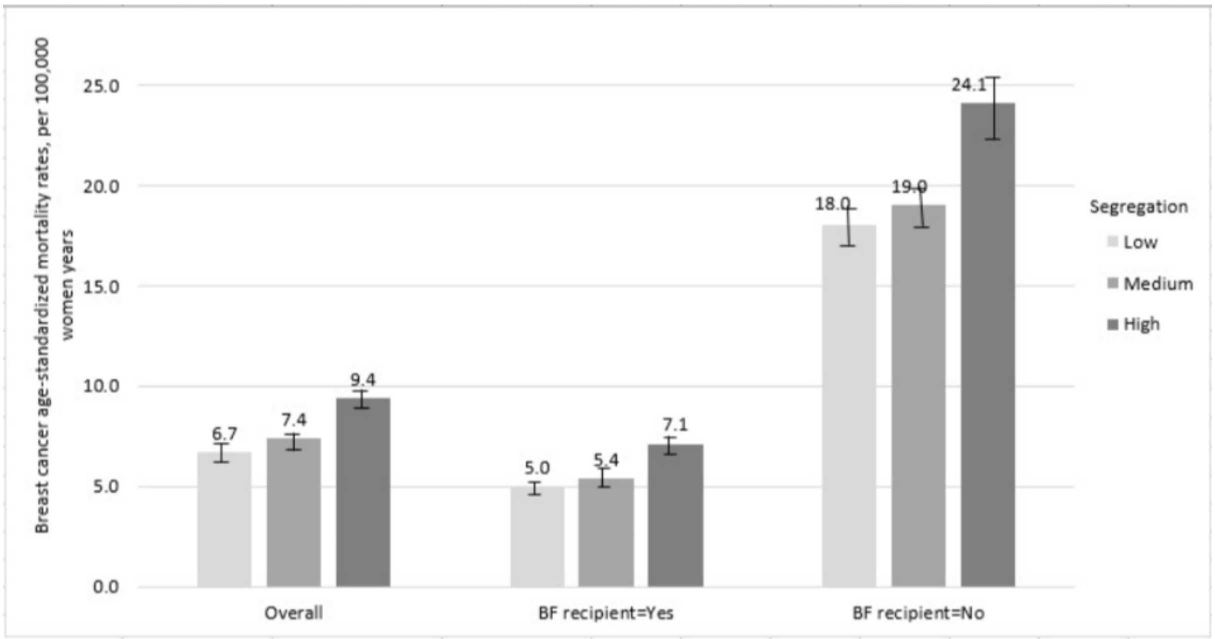

Note: BFP, Bolsa Família Programme. Income segregation measured using the dissimilarity index in tertiles: Low, Medium, High.

[a] Rates were age-standardized in 5-year age groups except for ages >=70 years which were collapsed into a single category, using the Segi's world population.

[b] Data are from the 100 Million Brazilian Cohort (2004-2015), n=21,680,930 women aged 18-100 years.

© 2023 Guimarães JMN et al. *JAMA Network Open.*

eTable 2. Mortality rate ratios (MRR) and 95%CI, obtained from sensitivity analysis to examine low death registration rate across Brazilian municipalities and the association between income segregation (in tertiles) and breast cancer mortality. [a]

	All municipalities n = 21,680,930 N of events = 15,387		Municipalities with death registration rate ≥95% n = 8,471,518 N of events = 7,583	
	MRR (95%CI), Crude	MRR (95%CI), Model 4	MRR (95%CI), Crude	MRR (95%CI), Model 4
Income segregation, Medium (vs Low)	1.12 (1.07,1.16)	1.08 (1.03,1.12)	1.01 (0.95,1.07)	0.98 (0.92,1.05)
High (vs Low)	1.45 (1.39,1.51)	1.18 (1.13,1.24)	1.26 (1.19,1.33)	1.14 (1.07,1.21)
Age at baseline	-	1.05 (1.05,1.05)	-	1.05 (1.05,1.06)
Race, Black (vs White)	-	1.10 (1.04,1.17)	-	1.24 (1.15,1.35)
Parda (vs White)	-	0.87 (0.84,0.90)	-	1.04 (0.98,1.09)
Asian (vs White)	-	0.73 (0.54,0.98)	-	0.93 (0.61,1.41)
Indigenous (vs White)	-	0.64 (0.45,0.90)	-	0.56 (0.30,1.05)
Education, 6-9 years (vs >9 years)	-	1.06 (1.00,1.13)	-	1.03 (0.95,1.12)
<= 5 years (vs >9 years)	-	0.98 (0.93,1.04)	-	0.91 (0.83,0.98)
Municipality size	-	1.00 (1.00,1.00)	-	1.00 (1.00,1.00)
Municipality population density	-	1.00 (1.00,1.00)	-	1.00 (1.00,1.00)
Area of residence, Rural (vs Urban)	-	0.70 (0.67,0.74)	-	0.77 (0.70,0.85)
Year of enrolment	-	0.98 (0.98,0.99)	-	0.99 (0.98,0.99)
Bolsa Família recipient, No (vs Yes)	-	1.17 (1.12,1.22)	-	1.11 (1.05,1.18)

Model 4: + Age + Race + Education + Municipality area size + Municipality population density + Area of residence + Year of enrolment + Bolsa Família Programme (BFP) recipient
Note: MRR, Mortality rate ratio. CI, Confidence interval. Income segregation measured using the dissimilarity index in tertiles: Low, Medium, High.
[a] Data are from the 100 Million Brazilian Cohort (2004-2015), n=21,680,930 women aged 18-100 years.

© 2023 Guimarães JMN et al. *JAMA Network Open.*
