# Supplementary material for: Income Segregation, Conditional Cash Transfers, and Breast Cancer Mortality Among Women in Brazil
Source: JAMA Netw Open. 2024 Jan 25;7(1):e2353100. doi: 10.1001/jamanetworkopen.2023.53100 (PMC10811554; doi:10.1001/jamanetworkopen.2023.53100)
Supplement: Supplement 2. — Data Sharing Statement [file jamanetwopen-e2353100-s002.pdf]

## Data Sharing Statement

Guimarães. Income Segregation, Conditional Cash Transfers, and Breast Cancer Mortality Among Women in Brazil. *JAMA Netw Open*. Published online January 25, 2024. doi:10.1001/jamanetworkopen.2023.53100

### Data
**Data available:** No

### Additional Information
**Explanation for why data not available:** All data supporting this study were obtained from the Center for Data and Knowledge Integration for Health (CIDACS). Data will be shared upon reasonable request to CIDACS and approval from the Ethics Committee.
